# Intraoperative evaluation of cervical nerve root avulsion using ultra–high-frequency ultrasound system

**DOI:** 10.1080/23320885.2019.1583569

**Published:** 2019-04-02

**Authors:** Daniel Boczar, Antonio J. Forte, Jeremie D. Oliver, Robert L. McClain, Peter Murray, Steven R. Clendenen

**Affiliations:** a Division of Plastic Surgery and Robert D. and Patricia E. Kern Center for the Science of Health Care Delivery, Jacksonville, FL, USA;; b Department of Anesthesiology and Perioperative Medicine, Jacksonville, FL, USA;; c Department of Orthopedic Surgery, Mayo Clinic, Jacksonville, FL, USA;; d Mayo Clinic School of Medicine, Mayo Clinic, Rochester, MN, USA

**Keywords:** Brachial plexus, ultra–high-frequency, ultrasound, peripheral nerve, spinal cord, plastic surgery, innovation, technology, microsurgery, imaging

## Abstract

Avulsion of C5 and C7 nerve roots was confirmed intraoperatively in a 21 year old male presenting after motor vehicle accident with confirmed absence of somatosensory evoked potentials upon stimulation via ultra–high-frequency ultrasound (70 MHz). Ultra–high-frequency ultrasound can be used as a reliable tool to directly visualise nerve injury.

## Introduction

Cervical nerve root avulsion following trauma is a critical consequence of brachial plexus injury, as it can severely impair upper-limb function [1]. The variety of potential intraoperative findings prompt meticulous evaluation both before and during surgery [2,3]. Especially when there are associated vascular lesions as in subclavian or axillary artery trauma. These injuries, which are present in 10% to 25% of cervical nerve avulsion cases, may limit reconstructive options [2]. Although Maldonado et al [3] suggest treatment of this condition has advanced slowly over the last 45 years, the evolution of imaging options (i.e. computed tomography [CT], magnetic resonance imaging [MRI], and ultrasound) has improved clinical decision making and outcomes.

Ultrasound technology has expanded substantially over the past few years. It is as a clinical tool well established in many different areas of medicine, which has both diagnostic and therapeutic uses. Many advances are seen in the field through the advent of digital processing, real-time B-mode imaging, and high-frequency transducers (i.e. higher than 30 MHz) [4–6]. The Ultra–high-frequency ultrasound (UHFUS) is a novel technology that can generate frequencies of up to 70 MHz providing superior imaging—up to 30 μm of resolution—sufficient to establish a precise type and level of nerve injury [6,7]. While increasing the frequency improves the image quality, this carries a relative disadvantage of diminishing the pulse length, thus reducing tissue penetration and visualisation of deep structures [5,6]. As this is a novel technology, there are few descriptions of UHFUS use in the literature. However, UHFUS is likely to become as widespread a tool in hospitals as other bedside ultrasound devices, as well as a relevant tool to evaluate patients with complex nerve injuries like brachial plexus lesions pre- and intraoperatively.

We present a case in which UHFUS was utilised preoperatively and intraoperatively to confirm the existence of a cervical nerve root avulsion diagnosed on MRI, and to **incidentally** identify a previously unseen higher-level nerve root avulsion. To our knowledge, this is the first report of UHFUS use in brachial plexus injury. Written consent was obtained from patient to publish this report.

## Case

**Figure 1.**
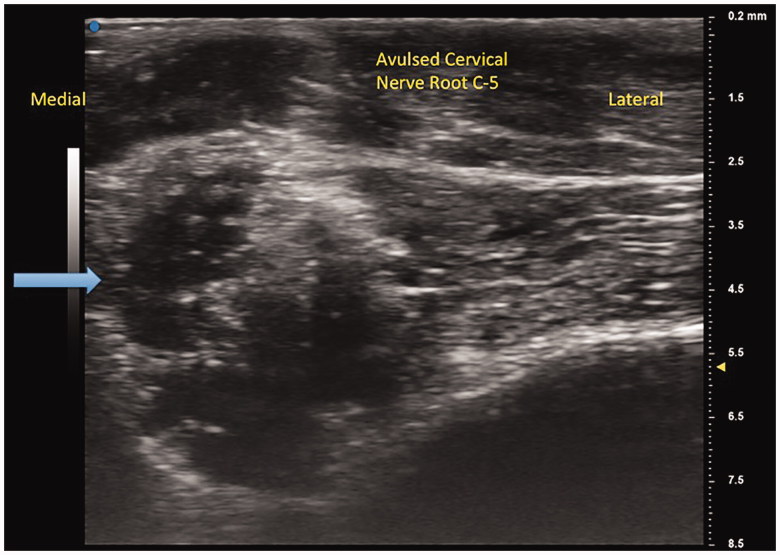
Avulsed cervical nerve root from C5 foramen. Blue arrow indicates separated nerve from the spinal cord.

**Figure 2.**
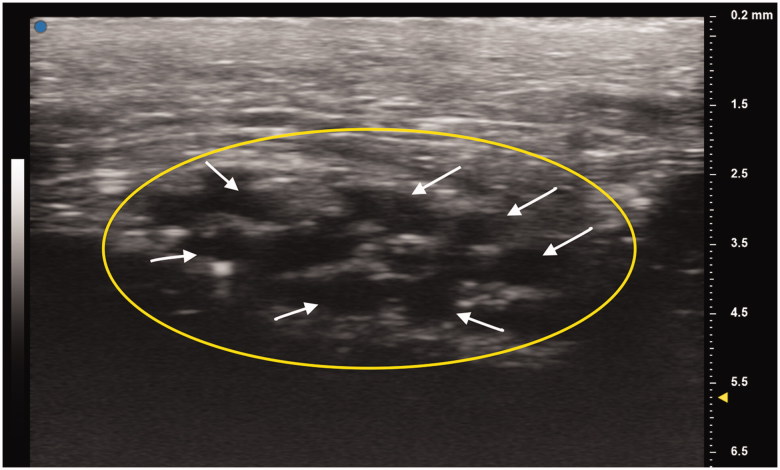
Patient’s unaffected left median nerve at the wrist crease. Oval indicates cross section of the median nerve. White arrows point to individual nerve fascicles.

**Figure 3.**
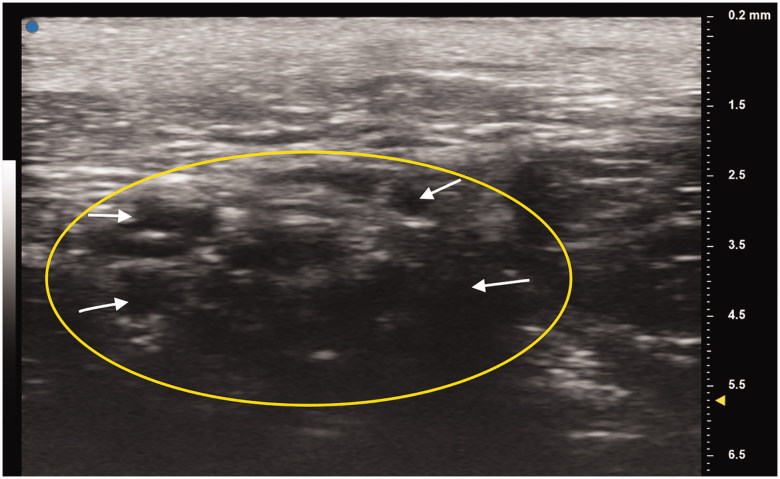
Patient’s deteriorated right median nerve at the wrist crease. Oval indicates cross section of the median nerve. White arrows point to individual nerve fascicles.

**Figure 4.**
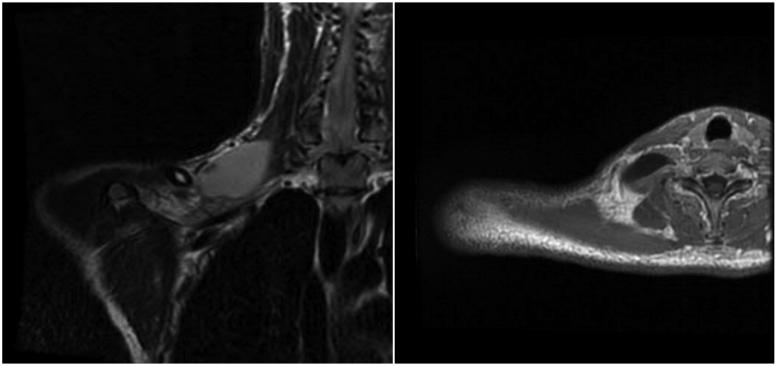
Patient’s magnetic resonance image showing a pseudomeningocele at C7 level. Coronal view on the left; axial view on the right.

A 21-year-old man with a history of traumatic right brachial plexus injury presented for brachial plexus exploration and possible nerve transfer after known avulsion injury of the C7 nerve root, resulting in loss of function in upper trunk innervated musculature, and incomplete recovery of C7-C8 nerve root innervated musculature function. Due to large pseudomeningocele obscuring the view of the nerve roots, C5 nerve avulsion was not detected on MRI. Preoperatively, bilateral UHFUS (70 MHz) images of the distal median nerves at the wrist were obtained. Intraoperatively, the brachial plexus was examined by placing the UHFUS probe directly on the roots as they exited the neural foramina to evaluate the degree of nerve avulsion from the spinal cord and feasibility of nerve transfer. In addition to confirmation of C7 avulsion, an avulsion of C5 was discovered and confirmed with absence of somatosensory evoked potentials upon stimulation, a diagnosis not originally made on MRI. A spinal accessory nerve-to-suprascapular nerve transfer and intercostal nerve transfers to the biceps branch of the musculocutaneous nerve were performed to restore neuromuscular function of the upper-limb (Figures 1–4).

## Discussion

The complexity of brachial plexus lesions requires a detailed evaluation before and during surgery, which is a challenge for medical teams [8]. The literature lacks a consistent management protocol for this condition [3]. CT and MRI occupy a crucial role in the preoperative evaluation, and careful surgical exploration with electrophysiological stimulation to analyse nerve function is utilised successfully intraoperatively [9,10] The ability of UHFUS to visualise smaller structures (such as peripheral nerve roots and branches) could contribute relevant information about these highly complex injuries, improving surgical strategy and decision making [11].

Interestingly, there are limited descriptions of UHFUS in the current literature regarding its application in humans, and even fewer studies about its use in nerve lesions. An author reported use of high-frequency probes to describe small cutaneous branches of nerves in the hands and fingers of cadavers and healthy volunteers and other author suggested UHFUS could be a new diagnostic tool for hand surgery after analysing 5 healthy volunteers and 1 patient with images obtained by surgeons who did not have formal training in ultrasound techniques [6,12]. Other applications where described such as Myredal et al [13], who demonstrated UHFUS to analyse intima-media thickness assessing atherosclerosis in hypertensive patients and also, Schneeberger et al, [14] who utilised UHFUS images of ulnar and radial arteries to monitor chronic graft rejection in a patient with hand allotransplant.

UHFUS is a potential new frontier of ultrasound technology, likely to become widely available in hospitals in the near future. In addition to being a non-invasive test, the **potential** cost benefit of ultrasound **has been discussed in the literature** when compared to other imaging modalities. In fact, a study performed across hospitals in Florida concluded that ultrasound has an average cost approximately one-fourth that of CT and one-fifth that of MRI [15]. Specifically in the field of hand surgery, ultrasound has been used for decades to evaluate the median nerve for carpal tunnel syndrome. An author pointed out, in a series of 195 patients with vague hand problems, that ultrasound findings influenced procedures and therapies in 21% of the cases [4]. This imaging technique, **while not novel to the field of hand surgery, could serve an innovative role** as a reliable tool to precisely evaluate complex nerve injuries, improve surgical planning and decision making **as an imaging adjuvant** in real-time, and reduce severe long-term deficits [5,8].

While this study is limited to a single-patient case report, it **demonstrates a novel application of the UHFUS technology to peripheral nerve surgery**, displaying its capacity to identify nerve root avulsion injuries, previously unrecognised on MRI. **While this report in no way advocates for complete substitution of the current standard imaging modality for brachial plexus lesions (neuro-MRI), this study does demonstrate promising applications of ultrasound technology as an adjuvant to diagnosis.** Additional human prospective studies are necessary to statistically support routine use of UHFUS in brachial plexus lesions, **and to compare cost burden of its utility to the current standard imaging modalities**.
